# Urothelial Carcinoma of the Bladder Metastatic to Bone Marrow Presenting as Isolated Thrombocytopenia

**DOI:** 10.1100/tsw.2007.168

**Published:** 2007-06-12

**Authors:** Robert C. Chan, Greg L. Hundemer, Amy Tatsas, Michael S. Cookson

**Affiliations:** ^1^ Department of Urology, Vanderbilt University Medical Center, Nashville, TN, USA; ^2^ Department of Pathology, Vanderbilt University Medical Center, Nashville, TN, USA

**Keywords:** urothelial carcinoma, thrombocytopenia, metastases, bone marrow, bladder cancer

## Abstract

The skeletal system is a frequent site for metastases of urothelial carcinoma (UC) of the bladder (22–37%). Of those cases involving bone, the marrow is infiltrated in 27% of patients. Imaging modalities, such as X-ray and CT, will detect gross skeletal lesions in the vast majority of these patients with bone marrow involvement, however, most patients with bone involvement are symptomatic at presentation. Additionally, there have been few reports in the literature of bone marrow metastases from UC presenting with isolated thrombocytopenia.

This case report describes the case of a 53-year-old male with muscle-invasive transitional cell carcinoma of the bladder treated with cystoprostatectomy. Preoperative evaluation was significant only for mild thrombocytopenia. Standard workup for metastatic bony involvement, which included history, physical, chest X-ray, and whole body CT, was negative. Postoperatively, the patient's thrombocytopenia worsened and he bled diffusely from his pelvic bed. Bone marrow biopsy was obtained and showed the entire marrow cavity to be filled with metastatic transitional cells.

In the event of a similar future presentation of isolated thrombocytopenia in the setting of invasive UC, the clinician should consider a bone marrow biopsy, in addition to the standard workup for metastatic bony involvement, prior to proceeding with any surgical intervention.

## CASE REPORT

An otherwise healthy, 53-year-old man presented with the recent onset of gross hematuria. Cystoscopy showed a sessile-appearing bladder wall mass. Transurethral resection of bladder tumor (TURBT) revealed a muscle-invasive urothelial carcinoma (UC) of the bladder. There was no evidence of excessive bleeding associated with this procedure or easy bruising preoperatively, thus no bleeding time assays were warranted. The bladder mass was approximately 4 cm in size, and was not palpable on examination under anesthesia (EUA). A standard workup for metastatic involvement included whole body CT and chest X-ray. CT scan showed questionable adenopathy in the pelvis, but no clear evidence of metastatic disease. Chest X-ray was negative. The patient had no complaints of bone pain and no history of recent fractures. On preoperative evaluation, his platelet count was significant for thrombocytopenia (80,000/µl). Practice guidelines suggest that this platelet level was suitable for surgery and did not command a preoperative hematology workup[1]. CBC, BMP, and coagulation studies were within normal limits. His preoperative medications included only nizatidine. The patient underwent cystoprostatectomy. The bladder tumor was staged T4a and measured 4 × 3.5 × 1.4 cm with histologic features of high-grade carcinoma. Six regional lymph nodes were found to be involved (T4aN2).

The postoperative course was complicated by worsening thrombocytopenia in addition to acute blood loss anemia secondary to diffuse bleeding from the pelvic bed. On postoperative day 1, his platelet count was 47,000/µl. A hematology consultation was obtained, however, his platelet count continued to fall to 37,000/µl on postoperative day 2 along with continued bleeding that required multiple transfusions of platelets and packed red blood cells. Following an unsuccessful trial of empiric steroids to try and improve on the platelet count, a decision was made to take the patient back to the operative suite for an attempt at identification of a correctable source of bleeding and/or to pack the pelvis. During the surgery, 1 l of blood was evacuated from his pelvis and diffuse pelvic bleeding was noted and packed.

On postoperative day 5, his platelet count was 31,000/µl. He continued to require regular platelet transfusions. His lowest platelet count of 13,000/µl occurred on postoperative day 7. The hematology service at that point proceeded with a bone marrow biopsy. Histologic examination (Fig. 1) revealed the entire marrow cavity filled with metastatic transitional carcinoma and fibrosis, establishing a diagnosis of stage IV (T4a N2 M1).

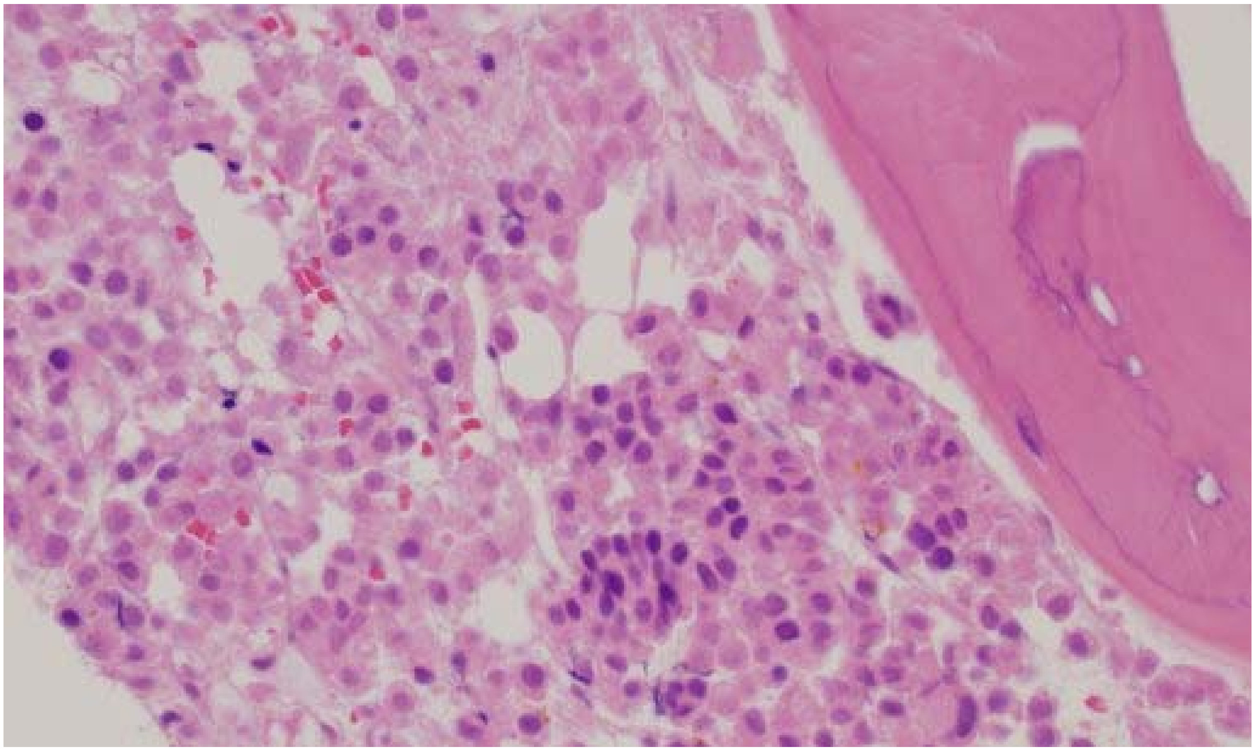

**FIGURE 1**. Bone marrow biopsy. H&E stain shows complete replacement of the bone marrow cavity with metastatic urothelial tumor cells, fibrosis, and tumor necrosis.

He was evaluated by medical oncology, but not considered a candidate for immediate systemic chemotherapy. This decision was made, in part, due to the poor response rate of individuals with visceral disease coupled with his significantly reduced performance status in the perioperative period. Furthermore, systemic chemotherapy likely would have further exacerbated his severe thrombocytopenia. A decision was made between the team and the family to treat the patient prophylactically with regular platelet transfusions to prevent further bleeding, and he was discharged to home after a 2-week hospitalization. In the event that the patient has some improvement in his clinical course, stabilizes his thrombocytopenia, and heals his abdominal wounds for a 4- to 6-week period, then re-evaluation for systemic therapy is warranted.

## DISCUSSION

Transitional cell carcinoma of the bladder frequently metastasizes to the lymph nodes (51–88%), liver (13–51%), lungs (36–40%), bone (22–37%), adrenal glands (6–21%), and rarely soft tissue and brain (2–6%)[2]. The workup of these patients for bone involvement includes chest X-ray, whole abdominal CT, and routine blood tests. Bone scans, once commonly used for staging in patients with known transitional cell carcinoma, are no longer recommended for asymptomatic patients as they are usually unable to identify patients with tumor stage T2 or greater who will not be cured by total cystectomy[3]. In cases of UC with bone metastases, 27% of patients have concurrent marrow infiltration[4]. However, most patients with bony involvement are symptomatic and/or have signs of widely metastatic disease. We presented a patient with UC of the bladder metastatic to bone marrow with corresponding thrombocytopenia without skeletal lesions on chest X-ray or CT.

It is extremely rare to find bone marrow involvement in the absence of radiographic evidence of skeletal lesions. In a study of 45 patients with bone metastases, a bone marrow biopsy was performed looking for UC tumor infiltration. Twelve patients were found to have bone marrow metastases, but only one lacked corresponding radiographic evidence of skeletal involvement[4]. A review of literature involving bone marrow metastases showed the lack of published reports of subsequent bone marrow suppression, such as thrombocytopenia, leukopenia, and anemia. Interestingly, the patient presented in this case displayed only thrombocytopenia (preoperative level of 80 thou/μl) while other indications of bone marrow failure were not present (e.g., white and red blood cell counts within normal limits). Though it is uncertain in this particular case why the platelets were the only indication of marrow failure, one possible explanation is that platelets have comparatively short life spans (10 days) compared with white (13–20 days) and red blood cells (120 days). Therefore, isolated thrombocytopenia in this patient may have been an indicator of early bone marrow dysfunction. This, to our knowledge, is the first report of a patient with UC metastatic to bone marrow without skeletal lesions on chest X-ray or CT clinically manifesting with thrombocytopenia.

Medication side effects leading to platelet count abnormalities or platelet dysfunction is a possible confounding cause that must be addressed. The patient's preoperative medications included only nizatidine, a $H_2$ receptor antagonist ($H_2R$). Although $H_2R$ antagonists ranitidine, famotidine, and cimetidine have been implicated in drug-induced immune thrombocytopenia lacking convincing laboratory documentation, fatal thrombocytopenia has been reported in only one patient treated with nizatidine and another $H_2R$ antagonist[5,6,7].

Solid tumors metastasize to bone by direct extension, retrograde venous flow, and seeding with tumor emboli via the blood circulation. The latter mechanism accounts for the vast majority of metastases to the red bone marrow where blood flow is high[8]. Once the tumor emboli reach the red marrow, they attach to stromal cells in the marrow by producing adhesive molecules[9]. The most common presenting symptoms of patients with bone marrow metastases were constitutional symptoms and bone pain. Signs of bone marrow involvement include anemia, thrombocytopenia, elevated red cell distribution width, and hypoproteinemia[10]. Once metastasis to the bone occurs, the prognosis is very poor, with a median survival of 6 to 9 months; few patients survive 5 years[11]. This case demonstrates the ability of highgrade UC of the bladder to metastasize to bone marrow, while escaping radiographic detection and causing such deleterious effects as thrombocytopenia. In the absence of symptoms, it is not routine clinical practice to obtain a bone scan in patients with muscle-invasive bladder cancer prior to radical cystectomy[3]. In hindsight, this may have been indicated, but the thrombocytopenia was discovered just prior to the surgery and not thought to be a harbinger of bone marrow infiltration. The authors recommend in the event of a similar future presentation for isolated thrombocytopenia in the setting of invasive UC the consideration of a bone scan and a bone marrow biopsy prior to proceeding with any surgical intervention.
